# The German Infant Nutritional Intervention Study (GINI) for the preventive effect of hydrolyzed infant formulas in infants at high risk for allergic diseases. Design and selected results 

**DOI:** 10.5414/ALX01462E

**Published:** 2017-08-04

**Authors:** A. von Berg, B. Filipiak-Pittroff, U. Krämer, E. Link, J. Heinrich, S. Koletzko, A. Grübl, U. Hoffmann, C. Beckmann, D. Reinhardt, C.P. Bauer, E. Wichmann, D. Berdel

**Affiliations:** 1 Forschungsinstitut, Klinik für Kinder- und Jugendmedizin, Marien-Hospital, Wesel; 2 Leibniz-Institut für umweltmedizinische Forschung (IUF), Düsseldorf; 3 Institut für Epidemiologie I, Helmholtz Zentrum München, Deutsches Forschungszentrum für Umwelt und Gesundheit, Neuherberg; 4 Dr. von Hauner’sches Kinderspital, Ludwig-Maximilian-Universität München; 5 Kinderklinik der Technischen Universität München, Germany

**Keywords:** primary allergy prevention, birth cohort, prospective, double-blind, randomised intervention study, hypoallergenic infant formula

## Abstract

In the complex interaction between certain environmental factors and genetic disposition, the early allergen exposure plays a major role in the development of allergic diseases. In aiming to reduce the allergen burden for the infant at risk during early infancy, cow’s milk protein hydrolysate infant formulas (hypoallergenic infant formulas) are appropriate alternatives to breastfeeding for primary allergy prevention. The **G**erman **I**nfant **N**utritional **I**ntervention-Program (GINI) was supported for the first 3 years by the German Ministry for Education and Research (BMBF) (FKZ 01 EE 9401-4). It is a birth cohort which was primarily scheduled until the children were 3 years old. The aim of the prospective, randomized, double-blind intervention study was to investigate the impact of different cow’s milk protein hydrolysate infant formulas in the first 4 – 6 months on the development of allergic diseases in children at risk due to at least one parent or biological sibling with a history of allergic disease. The allocation to one of the 4 intervention formulas (partial whey hydrolysate, extensive whey hydrolysate, extensive casein hydrolysate or standard cow’s milk formula) was randomised and stratified by family history (single/biparental) and the respective obstetric clinic. Recruitment was carried out by the three clinical centers (Research Institute Marien-Hospital Wesel, Children’s Department, Ludwigs-Maximilians-University Munich and Children’s Department Technical University Munich) in 18 obstetric clinics between 01.09.1995 and 30.06.1998. Along with the intervention study a non-interventional, complementary observational cohort of children with or without allergy risk was recruited and followed by annual self-reporting parental questionnaires. The GINI intervention study (GINI-I, N = 2.252) and the non-interventional observation study (GINI-NI, N = 3.739) are combined in the population-based GINIplus study (see article J. Heinrich et al. in this journal). The results of the GINI intervention study confirm that, cow’s milk protein hydrolysate infant formulas have a preventive effect on allergic manifestation compared with a standard cow’s milk formula, until school age. However, the dimension of the effect is different between the formulas. This effect, which is mainly driven by the effect on atopic eczema, develops in the first months of life and persists without rebound. In the formula groups the cumulative incidence of atopic eczema until school age is reduced between 26% and 45% compared with standard cow’s milk formula. A beneficial effect of the hydrolysate formulas on the respiratory manifestations asthma and rhinoconjunctivitis, however, could not be shown. By comparing the GINI intervention and non-intervention arm of the GINIplus study it was demonstrated, that a family history for allergy doubles the risk for eczema in the offspring. Early intervention with cow’s milk protein hydrolysate infant formulas is able to substantially compensate this risk for eczema until the age of 6 years. In contrast, by randomization to standard cow’s milk formula this risk showed a trend towards a higher incidence compared with children at risk from the non-intervention group. Thus, the results of the GINIplus study have contributed to answer some of the controversially discussed questions.

German version published in Allergologie, Vol. 35, No. 1/2012, pp. 32-43

## Introduction 

Allergic diseases develop as a result of the complex interaction between familial disposition and environmental factors that can either be protective or risk-enhancing. Among the known risk factors, early allergen exposure plays a decisive role. So far, interventional approaches have mainly aimed at reducing the allergen exposure to food and airborne allergens in early childhood in order to avoid sensitization and manifestation of the allergic disease. As newborn children have the most intensive allergen contact via food, most approaches to preventing allergies in infants with an increased risk for atopy are based on nutritional prevention with hypoallergenic baby food during the infant’s first months of life.[Table Abbreviations]

## Hypoallergenic baby food (cow’s milk protein hydrolysates) 

Hypoallergenic baby food means technologically treated formulas in which the milk proteins (casein or whey) are split to a varying extent by enzymatic cleavage, ultra heat treatment, and/or ultrafiltration. According to their degree of processing, hydrolysates are classified as either partial/weak (pHF) or extensive/strong (eHF) hydrolysates; depending on the original protein, they are also classified as either whey (W) or casein (C) hydrolysates. As a result, infant formulas with varying molecular weight profiles and varying residual antigenicity are available. 

Primary preventive nutrition with antigen-reduced hydrolysate formulas aims at developing oral tolerance in the child. This tolerance depends, among other things, on the structure and the dose or antigenicity of the allergen as well as on the child’s age at exposure. 

Based on data on the preventive effects of hypoallergenic infant formulas [1, 12, 22, 25, 29], hydrolysate formulas are recommended (since May 2011 also by the FDA) for not fully breastfed children with a family history of allergy [2, 3, 9, 13, 20]. Nevertheless, these recommendations are not only being questioned in part [8], but, in fact, a growing number of have been experts denying the primary preventive effects of partial hydrolysate in children at risk or even favoring exposure to intact cow’s milk protein during the first days of life to induce oral tolerance; this has lead to lively debates [14, 15, 16, 18]. These hypotheses must be proven or disproven in prospective controlled trials before the current recommendations can be changed [16]. 

## The history of GINI 

In the early 1980s, a scientific controversy arose over the use of weak or strong hydrolysate: which one would be better to avoid sensitization or to induce oral tolerance? 

From the clinical point of view, no answer to this question could be found. Due to the lack of studies on hypoallergenic infant formulas and on pHF vs. eHF or casein vs. whey [11, 21, 25], in 1985, when a special research field with the focus on allergy was announced by the BMBF, D. Berdel and A. von Berg applied for financial support for a comparative trial with different hydrolysate formulas.. This early project was supported by Prof. C.P. Bauer, TU Munich, and Prof. D. Reinhardt, LMU Munich, who both acted as clinical cooperation partners, and by Prof. H.E. Wichmann, Helmholz-Zentrum Munich (formerly GSF), who acted as a partner in the field of epidemiology. 

Although the application was positively appraised several times during the selection process that took several years, it was not finally approved until 1995. One of the reasons was that in the meantime the reunification of Germany had taken place and the money was needed for projects in former East Germany. On the other hand, all questions seemed to be solved and our approach seemed to be outdated by other studies, which had already been presented at meetings and were published later. These studies investigated hydrolysate vs. breast milk, cow’s milk, and weak hydrolysates as well as weak hydrolysate vs. a conventional cow’s milk formula [11, 21, 30, 31]. It was only when the reliability of earlier studies on hydrolysate nutrition was justly doubted (“Investigating the previous studies of a fraudulent author” [27]) that the BMBF remembered our application and granted financial support for 3 years in 1995. Thus, 10 years after the application had been initiated, we could finally start with our “investigation on the influence of nutrition during the first 4 – 6 months of life on allergy development in children with a family history of allergy and proven first-degree allergy exposure”, the so-called GINI (FKZ 01 EE 9401-4) and at the same time with the observation cohort (which received no financial support from BMBF) (see also article by Heinrich et al. in this issue). 

## GINI birth cohort 

The GINI birth cohort comprises the GINI intervention trial (GINI-I) and the non-intervention GINI observational trial (GINI-NI). 

## Design and method 

The prospective, randomized, double-blind intervention study GINI-I is the “German Infant Nutritional Intervention Program” to which the study owes its name. The aim of the study is the longitudinal investigation of early-childhood nutrition with three different cow’s milk protein hydrolysates compared with a regular cow’s milk-based infant formula with regard to allergy-preventive effects in children with familial allergy risk. 

By questionnaires on family history that were filled out by mothers before or immediately after delivery, a total of 2,252 healthy newborns (completed 37^th^ week of gestation; > 2,500 g) with at least one atopic family member could be included in the study (1,165 in the two Munich centers, 1,087 in Wesel). The participants were recruited in 18 obstetric wards between September 1, 1995 and June 30, 1998. The children were randomized to 1 of the 4 blinded study foods at their 14^th^ day of life at the latest, provided that they had not received any milk other than breast milk. 

Randomization was computer-controlled and stratified according to the region of the 18 participating obstetric hospitals (10 in Munich, 8 in Wesel and surroundings) as well as according to single or double atopy disposition in order to distribute known risk factors evenly to the 4 different study foods. The powdered milk was filled in identical cans with identical labeling, the only difference in the labeling being 1 code letter. A total of 4 code letters were used for each formula. Although the extensive hydrolysate formulas tasted different than the non or partially hydrolyzed formulas, allocation was not possible because 2 of the 4 formulas were extensively hydrolyzed. 

The 4 infant formulas used for the study were: the partial whey hydrolysate Beba-HA manufactured by Nestlé (pHF-W), the extensive whey hydrolysate Nutrilon Pepti manufactured by Nutricia, at the time marketed in Germany as HIPP-HA by the company HIPP (eHF-W), the extensive casein hydrolysate Nutramigen manufactured by Mead Johnson (eHF-C), and the conventional cow’s milk formula (CMF). The blinded study nutrition was provided free of charge for the first 6 months. 

The researchers recommended that the mothers breastfeed the children for 4 months, if possible. Only if the breast milk was insufficient were they recommended to feed the randomized study nutrition as the only milk nutrition during the 4-month strict intervention period. According to the then valid nutritional recommendations for children at risk for allergy, the parents were asked to introduce complimentary food during the child’s 6^th^ (or if necessary during the 5^th^) month of life; however, highly allergenic food such as eggs, dairy products, fish and nuts were recommended to be avoided until the end of the child’s 1^st^ year of life. Mothers were not recommended to adhere to an allergen-reduced diet. 

Parents were asked to keep a nutrition diary with weekly records in the first 6 months of the child’s life and monthly records in months 7 – 12. The records covered information on the form and amount of milk food (breast milk, study formula, mix), start and form of complementary food as well as the occurrence of skin reactions and other symptoms, like abdominal colics, vomiting, or diarrhea [4]. 

During their first 3 months of life, the children were examined for allergic diseases in the participating centers at certain time points (1^st^, 4^th^, 8^th^, 12^th^, 24^th^ and 36^th^ month of life). The physicians of the GINI team had received special training in the evaluation of allergic manifestations, including skin symptoms (SCORAD). Information on history of allergic symptoms and other diseases were obtained in interviews. Further questionnaires investigated risk factors for the development of allergies originating from the living situation as well as parents’ smoking behavior and sociodemographic factors. When allergic manifestation was suspected, the child was presented to an experienced allergy expert who made the final diagnosis as a so-called blinded observer. At the age of 4, 12, and 36 months, blood samples were drawn from the children to determine total and specific IgE. The specific child nutrition allergens (α-lactalbumin, β-lactoglobulin, casein, albumen, soybean) and airborne allergens (Dermatophagoides pter., Dermatophagoides farinae, cat dander, timothy grass, birch) were determined at 12 and 36 months; at the age of 4 months, only the three protein allergens (α-lactalbumin, β-lactoglobulin, casein) were determined [5]. 

According to the agreement with the project-executing organization, the prospective observation period was stipulated to be the first 3 years of each child’s life. It was, however, the plan to continue the study beyond this age. 

The study protocol was approved by the local ethics committees of the participating centers. Written informed consent was obtained from the parents. The GINI study as well as the follow-up examinations of the entire GINIplus cohort at the age of 6 and 10, including genetic investigation, were separately approved by the ethics committees. 

Together with the 2,252 children of the GINI intervention study (GINI-I), further 3,739 children were recruited for the GINI non-intervention study (GINI-NI) in the same obstetrics departments in Wesel and Munich between January 1996 and June 1998 using identical questionnaires to investigate family history for atopy. The GINI-NI cohort was composed of 1,232 children with, and 2,507 children without, a family history of atopy. The grouping into the GINI-I or the GINI-NI study was carried out according to familial atopy risk and the parents’ willingness to let their child participate in the prospective randomized double-blind intervention study. GINI-NI is a pure observational study to assess the natural course of the child’s development, i.e., no recommendations were made by the study investigators, particularly not with regard to a certain diet, so that mothers could feed their child whatever they felt appropriate, including hydrolysate nutrition [7]. 

Both study arms of the GINI cohort are summarized under the term GINIplus (N = 5.991). All parents (including the parents of the GINI-I children and in addition to the other interventional measures) received identical extensive questionnaires at their child’s 1^st^, 2^nd^, 3^rd^, 4^th^, 6^th^ and 10^th^ birthday. These questionnaires covered information on the child’s illnesses, nutritional habits, various environmental influences (e.g., traffic density, pet keeping, passive smoke exposure), and socioeconomic parameters. At the age of 6 and 10 years, all children were invited to the study centers for clinical examination, including the taking of blood samples (Table 1 in Heinrich et al., this issue). 

The strength of the two study arm design is that GINIplus is a population-based birth cohort that allows for the comparison of the interventional and the non-interventional study arm and this can help solving the question of allergy prevention by hydrolysate nutrition. Thus, it is of particular interest to find out how children who are at risk of an allergy develop in the intervention group as compared to children with an allergy risk in the non-intervention group. 

## Results of the GINI intervention study until the 6^th^ year of life 

### Study population

By stratified randomization, we made sure that the subjects were equally distributed between the 4 food groups and according to the previously known risk factors (sociodemographic differences, single/double family history of allergy) [4]. 

An early high dropout rate in the eHF-C group due to refusal of food (mainly because of the taste) was compensated by further randomization. 889 mothers who complied with the recommendations to exclusively breastfeed their child were equally distributed between the nutritional groups. The first analysis at the age of 12 months included only children who had received the recommended milk nutrition (only the randomized formula with or without breastfeeding) during their first 4 months of life (strict intervention phase), i.e., exclusively breastfed children, dropouts, and children who were non-compliant with the milk nutrition [26] were excluded (per-protocol (PP) analysis). The remaining children were also equally distributed between the study groups. At the age of 3, 6, and 10 years, an intention-to-treat (ITT) and a PP analysis were carried out, with stronger results in the latter one because the ITT groups comprised about 50% of children who were exclusively breastfed. 

Until and including the 3-year examination, the results were based on a combination of the data derived from the interviews and the clinical findings, thereafter they were based on the evaluation of the parents’ answers in the annual questionnaires to the following questions: “Has a physician ever / in the past 12 months / since the last survey diagnosed an allergic disease like atopic dermatitis / eczema / neurodermitis, asthma or hay fever / allergic rhinoconjunctivitis, food allergy, allergic urticaria?”. Simultaneously, symptoms of these diseases and corresponding drug use were inquired. 

### Selected results of nutrition with various cow’s milk protein hydrolysates on the development of allergic diseases in children with familial allergy risk

**Figure 1:**
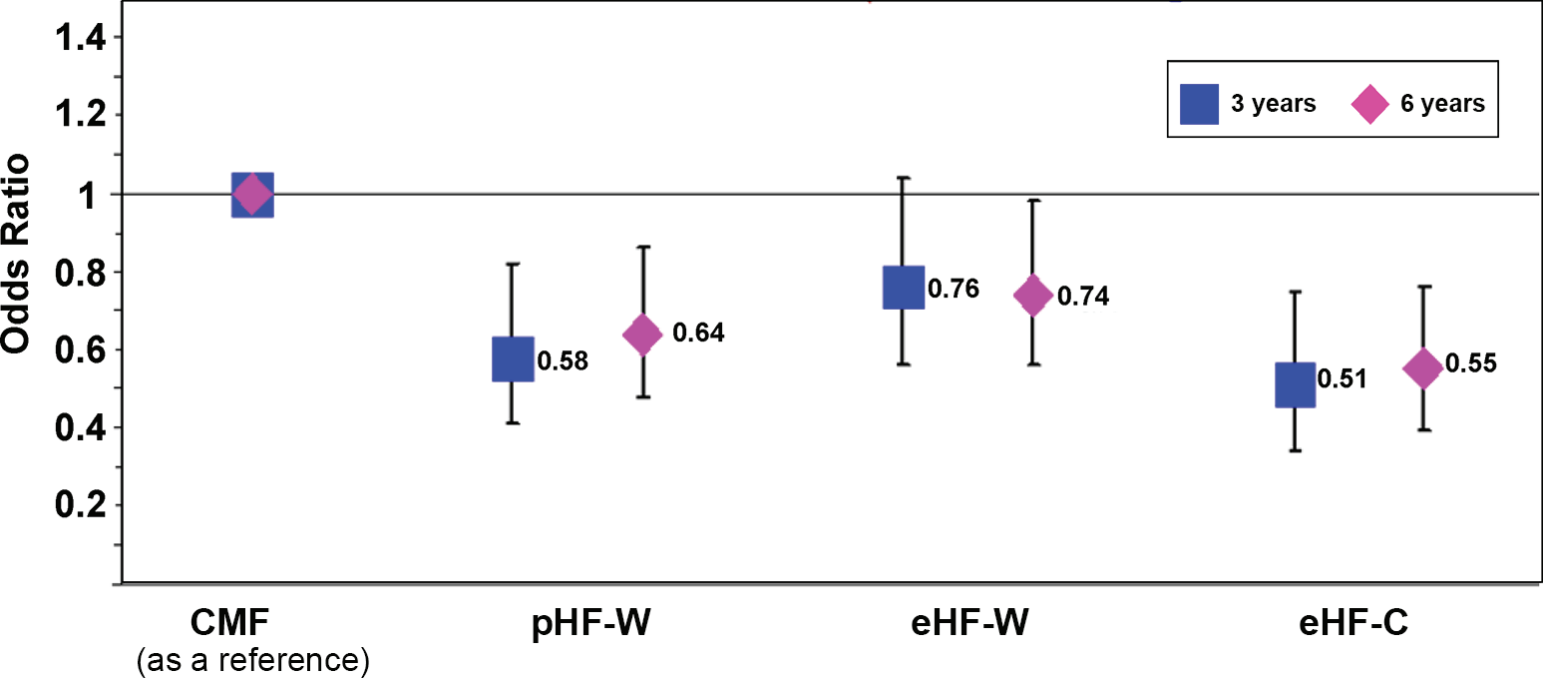
Cumulative incidence. Eczema at the age of 3 and 6 years in the GINI intervention study. CMF = standard cow’s milk formula; pHF-W = partial whey hydrolysate; eHF-W = extensive whey hydrolysate; eHF-C = extensive casein hydrolysate.

Milk protein hydrolysates (hypoallergenic baby food) have a protective effect (as compared to non-hydrolyzed baby food based on intact cow’s milk proteins) on the development of allergic manifestation until the age of 6 years, which is mainly due to the reduction of atopic eczema (Figure 1) [4, 5, 6]. The preliminary evaluation of the 10-year examination shows that the results of the 6-year examination are being continued. Thus, GINI is the first study to demonstrate that the early effects are maintained until school age. This finding puts an end to the frequently asked question on a rebound effect and demonstrates that the prevention is persisting and that the onset of the disease is not only delayed (submitted for publication). 

We could confirm results obtained by other authors [30, 31] who show that the protective effect of hydrolysate nutrition only develops within the first months of life [4]. The impact of this finding on the clinical use of preventive measures with hydrolysate nutrition is important because the time frame in which an effect can be expected (so-called window of opportunity) is limited to the first 4 – 6 months of life. 

The dimension of the preventive effect of individual hydrolysates on atopic manifestations varies and is, in part, modified by the individual genetic risk of the child [4]. For example, in the 1^st^ year of life, we could observe an effect of the weak whey hydrolysate of more than 50% only in children whose parents did not suffer from atopic eczema (but from asthma or allergic rhinitis), while the strong casein hydrolysate reduced the child’s risk of developing eczema to the same degree, independently of the familial risk. Nevertheless, the differences between the two hydrolysates were not significant. 

Until the GINI results were published, it was thought that the preventive effect of a milk protein hydrolysate depended on its molecular weight. Thus, the expected order of the effect of the foods used in GINI would have been: eHF-C, eHF-W, pHF-W. The results up to the 3^rd^ year show, however, that a preventive effect is only present for eHF-C and pHF-W. Only at the age of 6 years did we find a relatively weak, but statistically significant, reduction of the cumulative incidence of atopic dermatitis in 26% of children in the eHF-W group, while the cumulative incidence was 36% in the pHF-W group and 45% in the eHF-C group (Figure 1) [5, 6]. It has to be concluded that the effect of a hydrolysate neither depends on its basic protein (whey/casein) nor on the degree of hydrolyzation (partial, extensive) alone. This contradicts the opinion that the lower antigenicity of hydrolyzed baby food demonstrated in vitro is also responsible for the in-vivo clinical effect. Instead, what seems to be decisive is to which extent the antigenicity of the epitopes resulting from the hydrolyzation process is reduced. As this varies according to hydrolysate, this means that hydrolysates are not interchangeable, but that only hydrolysate nutrition whose preventive effect was shown in controlled clinical trials should be used for allergy prevention. 

Some earlier studies described a certain protective effect of hydrolysate nutrition on early wheezing. However, due to the relatively short observation period of this study [11, 21], this refers only to the first 2 – 3 years, in which wheezing frequently is not a sign of asthma onset but rather a symptom of viral obstructive ventilation disorders [28]. We therefore did not make a diagnosis of allergic asthma before the age of 3 years, based on a strict definition. A preventive effect of hydrolysate nutrition on asthma could not be observed in the GINI study. Likewise, hydrolysates did not, at any point in time, have a preventive effect on early wheezing or on the later wheezing, which is a cardinal symptom of allergic asthma.. In addition, an effect on allergic rhinitis could not be detected [6]. 

The incidences and prevalences we observed are low, which might best be explained by our strict diagnostic criteria [16]. 

Although the GINI study does not provide any information on the mechanism of action of hydrolysate nutrition, the lack of effect on asthma and allergic rhinitis might be interpreted as evidence for the fact that the development of respiratory allergic diseases cannot be influenced by a peroral preventive approach. 

## Further GINI results 

### Growth

In addition to the efficacy of hydrolysate nutrition, its safety with regard to a normal physical development of the children is decisive. Until the age of 6 and 10 years, no differences with regard to the growth of the children (based on BMI development) could be observed, neither between the children who received hydrolysate nutrition, nor when these children were compared to those who received cow’s milk formula and/or breast milk. However, a significantly lower BMI development was observed in the children of the eHF-W group in the months 4 – 48 as compared to the other groups, and this was only due to the lower body weight of the children in the eHF-W group. Whether this finding will have effects later in life, needs to be investigated in further analyses. 

### Cost effectiveness

Another important aspect of primary prevention with hydrolysate nutrition is its cost effectiveness. The analysis of the GINI data obtained until the 6^th^ year of life showed that the use of pHF-W and eHF-C during the first 4 months of life is not only cost-effective but also cost-saving [19]. 

### Development of taste

There is evidence suggesting that the use of hydrolysate nutrition in the first months of life influences later taste preferences; nevertheless, it is not known how long this early programming lasts. In a subgroup of children, a preference test was carried out at the age of 10 years and it could be shown that early feeding with one of the three hydrolysate foods was significantly associated with a higher acceptance of the strong casein hydrolysate as an example of bitter taste [24]. 

## Selected results of the GINIplus study until the 6^th^ year of life 

### Study population

The joint analysis of the two study arms is possible due the identical annual questionnaires. Of the 5,991 subjects, a total of 3,833 (64%) participated in the 6-year survey: 2,153 (57.6%) in the GINI observation study and 1,680 (74,6%) in the GINI intervention study [7]. 

### Impact of nutrition with hydrolysate food in the GINIplus cohort

**Figure 2:**
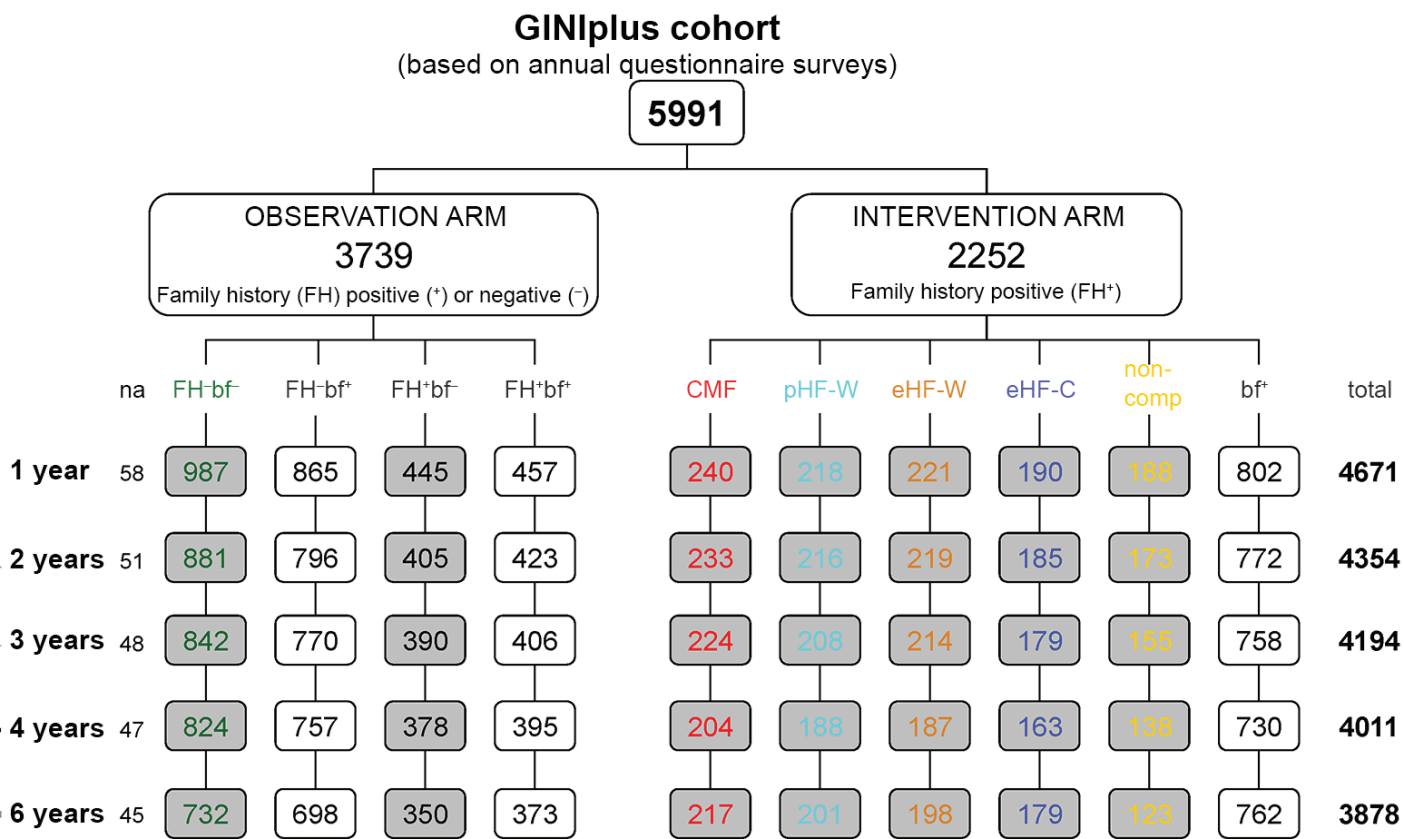
GINIplus birth cohort until the age of 6 years; questionnaires for expert diagnosis of allergic diseases answered by parents. FH^+^ = positive family history of allergy; FH^–^ = negative family history of allergy; bf^+^ = exclusively breastfed during the first 4 months; bf^–^ = formula nutrition used exclusively or in addition to breastfeeding during the first 4 months of life, with the formula being freely selectable by parents in the non-intervention study; CMF = standard cow’s milk formula; pHF-W = partial whey hydrolysate; eHF-W = extensive whey hydrolysate; eHF-C = extensive casein hydrolysate; non-comp = non-compliant with milk nutrition; na = no information on nutrition available.

For a better illustration of the comparison between GINI-I and GINI-NI and the development of atopic dermatitis in the different populations, we differentiated between 10 groups (Figure 2). 

GINI-I is subdivided into 6 groups: 4 groups for the different formulas used (CMF = regular cow’s milk formula, pHF-W, eHF-W, and eHF-C), 1 group for the ‘non-compliants’ (with milk nutrition in the first intervention phase = non-comp), and 1 group for children who were exclusively breastfed (bf^+^). All children in GINI-I have a positive family history for atopy (FH^+^). 

GINI-NI is subdivided into 4 groups: with or without atopic family history (FH^+^, FH^–^), and these either exclusively breastfed (bf^+^) or fed with formula nutrition at the parent’s discretion (bf^–^). 

Separate analyses were performed for subjects in the NI group who were fed with a formula of the parent’s choice for the first months of life and for children who were exclusively breastfed for the first 4 months of their lives. 

**Figure 3:**
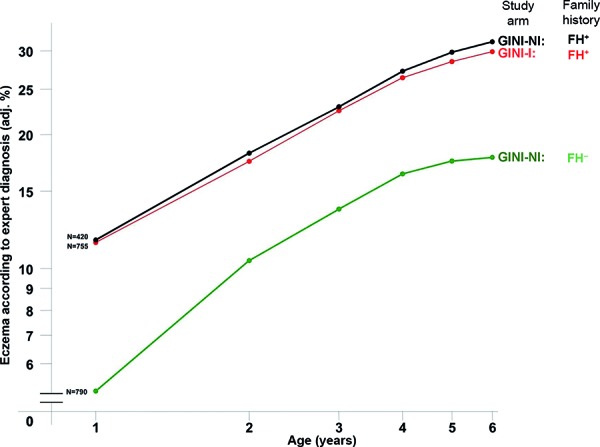
Cumulative incidence of atopic eczema in children with (FH^+^) and without (FH^–^) allergy risk who were exclusively breastfed during their first 4 months of life in the GINI intervention study (I) and in the non-intervention observational study (NI).

**Figure 4:**
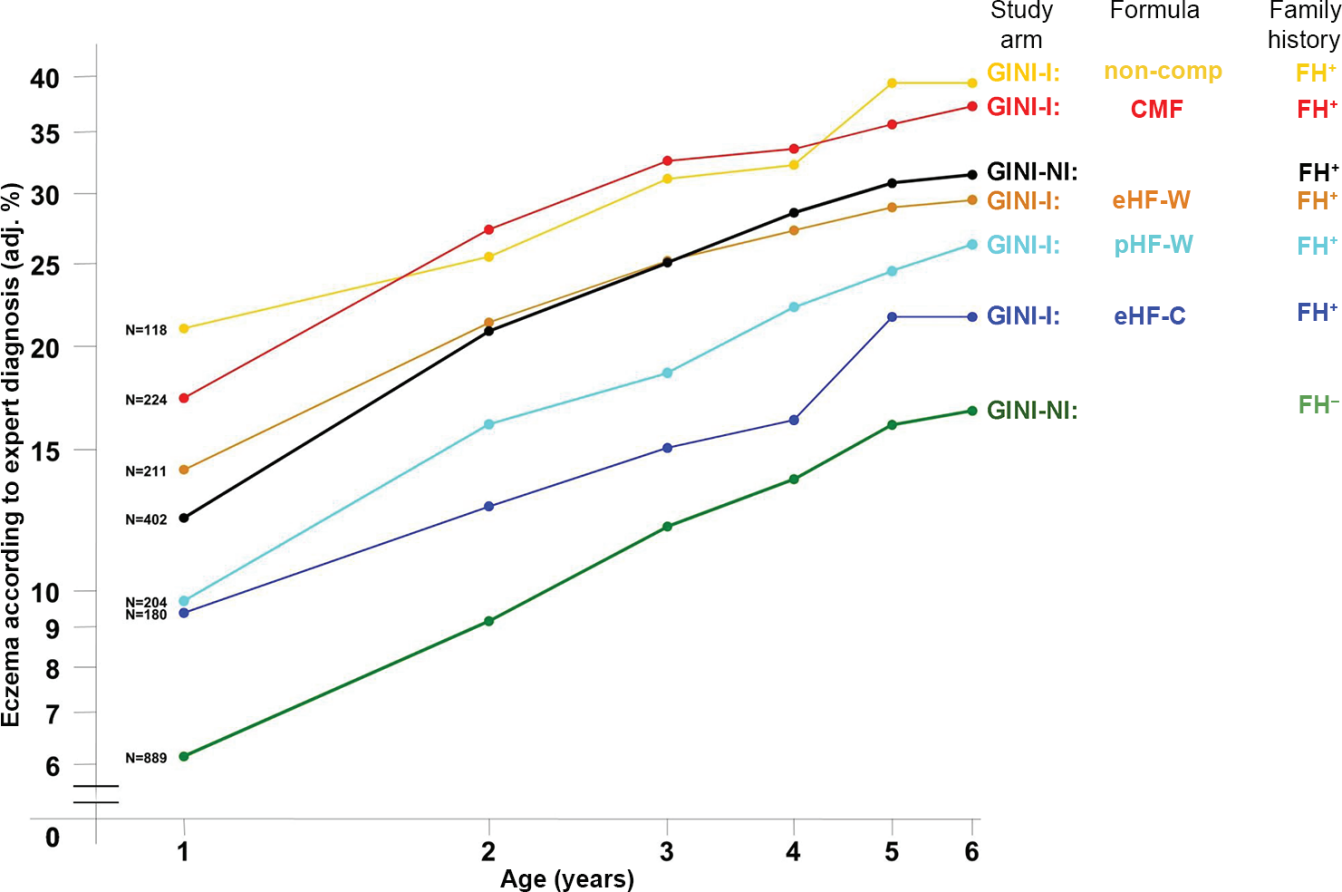
Cumulative incidence of atopic eczema in children with (FH^+^) and without (FH^-^) allergy risk in whom formula nutrition was used exclusively or in addition to breast milk during their first 4 months of life in the GINI intervention study (I) and in the non–intervention observational study (NI). CMF = standard cow’s milk formula; pHF-W = partial whey hydrolysate; eHF-W = extensive whey hydrolysate; eHF-C = extensive casein hydrolysate; non-comp = non-compliant with milk nutrition.Figure 1. Cumulative incidence. Eczema at the age of 3 and 6 years in the GINI intervention study. CMF = standard cow’s milk formula; pHF-W = partial whey hydrolysate; eHF-W = extensive whey hydrolysate; eHF-C = extensive casein hydrolysate.

It was shown that the risk of developing atopic eczema was twice as high in children with a positive family history for atopy, regardless of whether they received any one of the formula foods (OR 2.1 (95% CI 1.6 – 2.7)) or were exclusively breastfed (OR 1.9 (95% CI 1.5 – 2.4)) (Figure 3, Figure 4). 

To evaluate the influence of the interventional measures taken apart from the different study foods in the children’s 1^st^ years of life, the fully breastfed GINI-I and GINI-NI children with positive family history were further analyzed. 

The almost identical course of the cumulative incidence of atopic eczema (Figure 3) shows that the interventional measures in the GINI-I group taken in the 1^st^ year of life (nutritional advice, regular support by the GINI team including clinical examinations, diary keeping) did not have any influence. 

Under these circumstances it was possible to compare the formula-fed children of GINI-I with those in GINI-NI. The GINI-NI group who had a negative family history and was fed with formula nutrition (NI, FH^–^bf^–^) was used as a reference group. It could be shown that an early intervention with hydrolysate nutrition can compensate for the doubled risk of FH^+^ children developing eczema to a varying extent up to their 6^th^ year of life – and an extensive casein hydrolysate can even the risk out to a non-significant difference – while a regular cow’s milk formula increases the risk (Figure 4) [7]. This result underlines the importance of hydrolysate nutrition in children at risk, if the breast milk does not suffice during the first 4 months of life. 

Results on the **influence of breastfeeding** [17] and on the time of introduction and variability of **solid food** [10] are presented in Heinrich et al., this issue. 

## Final remarks 

With 2,252 children, the GINI intervention study is the largest trial ever carried out with regard to primary allergy prevention using various hydrolysate formulas. Furthermore, it is the trial with the longest follow-up period (10 years completed). It should especially to be highlighted that it is independent of companies manufacturing baby food. 

The 15-year follow-up of the GINIplus cohort started at the end of 2010. Further interesting results can be expected. 

**Abbreviations. Abbreviations:** 

GINI: **G**erman **I**nfant **N**utritional **I**ntervention-Program
GINI-I: GINI intervention study
GINI-NI: GINI non-intervention observational study
GINIplus: GINI-I plus GINI-NI
FDA:****Food and Drug Administration
pHF-W: partial or weak whey hydrolysate
eHF-W: extensive or strong whey hydrolysate
eHF-C: extensive casein hydrolysate
CMF: standard cow’s milk formula
FH^+^: positive family history
FH^–^: negative family history
ITT: intention-to-treat analysis
PP: per-protocol analysis
bf^+^: exclusively breastfed during the first 4 months of life
bf^–^: formula nutrition used exclusively or in addition to breastfeeding during the first 4 months of life, with the formula being freely selectable by parents in the non-intervention study
non-comp: non-compliant to the recommended mild nutrition in the intervention group
95% CI: 95% confidence interval
na: not available
